# A 77 GHz Power Amplifier with 19.1 dBm Peak Output Power in 130 nm SiGe Process

**DOI:** 10.3390/mi14122238

**Published:** 2023-12-14

**Authors:** Peigen Zhou, Pinpin Yan, Jixin Chen, Zhe Chen, Wei Hong

**Affiliations:** State Key Laboratory of Millimeter Waves, School of Information Science and Engineering, Southeast University, Nanjing 210096, Chinaweihong@seu.edu.cn (W.H.)

**Keywords:** DAC, power amplifier, SiGe, W-band

## Abstract

This article reports a two-stage differential structure power amplifier based on a 130 nm SiGe process operating at 77 GHz. By introducing a tunable capacitor for amplitude and phase balance at the center tap of the secondary coil of the traditional Marchand balun, the balun achieves amplitude imbalance less than 0.5 dB and phase imbalance less than 1 degree within the operating frequency range of 70–85 GHz, which enables the power amplifier to exhibit comparable output power over a wide operating frequency band. The power amplifier, based on a designed 3-bit digital analog convertor (DAC)-controlled base bias current source, exhibits small signal gain fluctuation of less than 5 dB and saturation output power fluctuation of less than 2 dB near the 80 GHz frequency point when the ambient temperature varies in the range of −40 °C to 125 °C. Benefiting from the aforementioned design, the tested single-path differential power amplifier exhibits a small signal gain exceeding 16 dB, a saturation output power exceeding 18 dBm, and a peak saturation output power of 19.1 dBm in the frequency band of 70–85 GHz.

## 1. Introduction

In recent years, with the increasing attention paid to driving safety and intelligent driving, autonomous driving and advanced driving assistance systems have gradually become the focus of people’s attention. Autonomous driving (assisted driving) mainly uses on-board sensors to sense the vehicle’s surroundings and, based on the information obtained about the road, vehicles, and obstacles, to then control the vehicle’s steering and speed, making driving safer [1]. Therefore, high-precision and high-sensitivity sensors are among the core devices in assisted driving systems.

Millimeter wave radar has been widely used in assisted driving due to its advantages of high accuracy, good real-time performance, strong environmental adaptability, and so on [2]. In the 76–81 GHz band, in-vehicle millimeter wave radars are able to use bandwidths in excess of 3 GHz for front-end long-range radars for applications such as adaptive cruise control and corner radars for applications such as emergency braking. As one of the core devices in the millimeter wave radar system, the output power of the power amplifier restricts the performance of the transmitter, which, in turn, determines the wireless range of the radar system.

III–V compound semiconductor processes are widely used in the design and fabrication of amplifiers in the 77 GHz band because of their higher cutoff and maximum oscillation frequencies, higher cutoff voltages, and lower substrate losses [3]. However, the III–V process has a lower level of chip integration due to fewer metal layers [4]. Due to the advantages of the silicon-based process in terms of integration, yield, and cost, coupled with the continuous improvement of the output power performance of the silicon-based process in the 77 GHz band, 77 GHz power amplifiers developed based on the silicon-based process have been continuously reported [5,6,7]. For the power amplifier in a 77 GHz vehicle radar system, a high-output power is required in order to have a long range of action for the radar [8,9]. In order to meet the imaging requirements, multiple transmitter channels need to be integrated, which puts severe requirements on the number of integration paths and chip size of the power amplifier. In addition, automotive regulations require that on-board millimeter-wave radars operate normally over −40 °C to 125 °C, so the power amplifier needs to maintain high output power and gain stability over −40 °C to 125 °C temperature range. Due to the high operating frequency and large path loss in the 77 GHz frequency band, as well as the limited RF output power of semiconductor technology, multiple RF channels need to be integrated on the chip to achieve beamforming control and improve the effective isotropic radiated power (EIRP) of the transmitter in order to achieve longer detection distances. Moreover, the size of each channel needs to meet the requirements of array integration, which places higher demands on the size of the power amplifier.

In this paper, a power amplifier with an operating center frequency of 77 GHz is designed based on a 130 nm SiGe process. Benefiting from the amplitude and phase balance tuning capacitor introduced at the center tap of the Marchand balun’s secondary coil, this power amplifier exhibits excellent RF performance over a wide operating band. By using a symmetrically connected, multiple transistors in parallel architecture in the power amplifier stage, the peak output power of the single-path differential power amplifier reaches 19.1 dBm, avoiding the need for a multiplexed power synthesis architecture that requires a larger chip size. Based on a current source controlled by an on-chip integrated 3 bit DAC, the power amplifier maintains good output power and small-signal gain over fluctuations in ambient temperatures ranging from −40 °C to 125 °C. The organization of this article is as follows: Section 2 focuses on introducing the design method of the 77 GHz power amplifier; Section 3 presents the experimental results of the power amplifier; and Section 4 concludes with a brief summary.

## 2. Circuit Design

As the nodes of the silicon-based process continue to shrink, the cut-off frequency of the transistors continues to increase, making it possible to implement higher-frequency amplifiers based on silicon-based process designs [10]. However, as the cut-off frequency increases, the withstand voltage value of the silicon-based process transistor decreases, so the supply voltage is limited, which, in turn, affects the output power of a single transistor. In addition, the silicon-based process has a high substrate dielectric loss, which affects the quality factor of the on-chip passive components and increases the loss of the passive components, further limiting the output power of the silicon-based power amplifier [11,12]. 

A common approach to boosting the output power of power amplifiers is to use power synthesis techniques, including on-chip synthesis and spatial synthesis [13,14]. On-chip power synthesis is used to boost the output power of an amplifier by designing a passive synthesis network inside the chip to sum the output power of multiple channels with high efficiency. Spatial power synthesis is carried out by controlling the radiation direction of each transmitting antenna array, which, in turn, synthesize the power of the amplifiers in space according to a certain phase. Since 77 GHz radars require a multi-channel architecture for angle scanning, on-chip multiplexed power synthesis or spatial power synthesis is often unsuitable for power amplifiers applied in radar systems due to the large chip area required.

**Figure 1:**
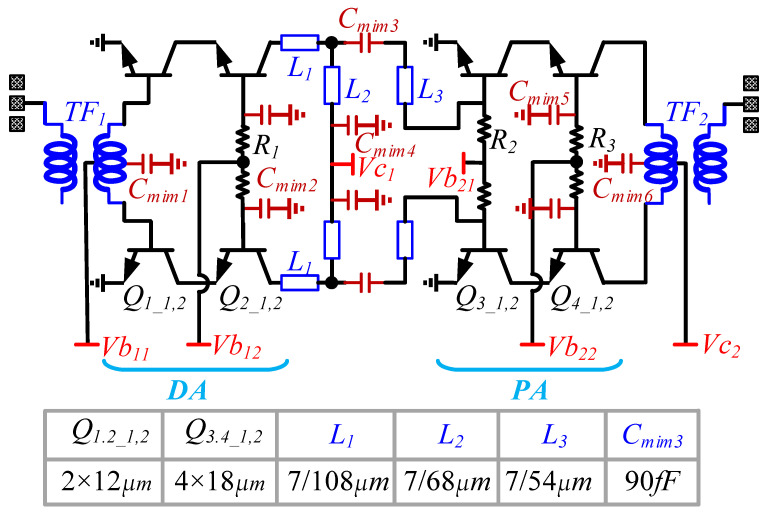
Schematic of the 77 GHz PA.

The circuit schematic of the power amplifier is shown in Figure 1, which includes an input Marchand balun, a differential common emitter-common base (cascode) topology driver amplifier (DA), an inter-stage impedance matching network, a differential cascode structure power amplifier (PA), and an output Marchand balun. In order to effectively enhance the output power of the power amplifier, four transistors with an emitter length of 18 microns are connected in parallel in the design of the PA stage to enhance the power capacity of the transistors, which, in turn, enhances the output power of the power amplifier. The peak saturated output power of this single-path differential power amplifier exceeds 19 dBm in the 77 GHz band when power synthesis techniques are not employed.

Unlike conventional Marchand baluns, an on-chip tuning capacitor *C*_*mim*1_ is introduced at the center point of the secondary coils that make up the balun, allowing the balun to maintain good amplitude and phase balance characteristics over a wide frequency band. Due to the large difference between the output impedance of the DA stage and the input impedance of the PA stage, the use of a single-turn transformer for intermediate impedance matching has a large loss and a narrow bandwidth. The use of a 1:*n* multi-turn transformer can achieve a better inter-stage impedance matching, but the design of a 1:*n* multi-turn transformer is more difficult. Therefore, the inter-stage matching is implemented using a broadband multi-branch network, as shown in Figure 1, including series microstrip lines *L*_1_ and *L*_3_, series capacitor *C*_*mim*3_, and parallel microstrip line *L*_2_. Output impedance matching is similar to input impedance matching and is implemented using Marchan balun. The size of the DA stage’s transistor is 2 × 18 μm, the size of the PA stage’s transistor is 4 × 18 μm, and the parameters of the key passive devices are illustrated in Figure 1. 

### 2.1. Design of the Broadband Marchand Balun

**Figure 2:**
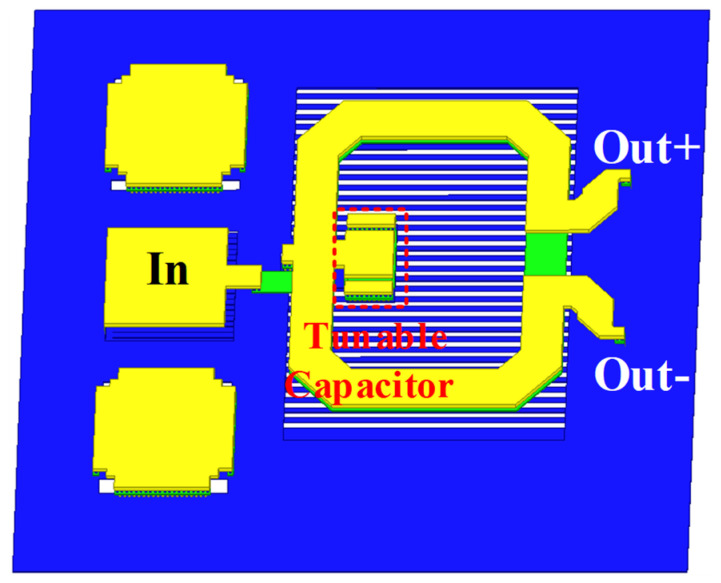
3D layout of the Marchand balun with input radio frequency (RF) PAD.

The power amplifier uses a differential structure, but for the convenience of testing, the power amplifier adopts single-ended input and output. Therefore, a balun for converting differential signals to single-ended signals is inevitably required at the input and output terminals of the power amplifier. Marchand baluns are widely used in single-ended-to-differential signal conversion networks in the millimeter wave band. However, when the operating frequency rises to the E-band (60–90 GHGz), it usually faces the problem of amplitude and phase imbalance. Both the input and output impedance matching network of this 77 GHz power amplifier are implemented using Marchand baluns. Unlike the conventional Marchand balun, we co-design the Marchand balun with the input (or output) RF PAD to fully consider the effect of RF PAD’s parasitic capacitance on the matching network. In addition, we introduce a tunable capacitor at the center tap of the secondary coil that forms the Marchand balun, as depicted in Figure 2. By tuning the capacitance value, the amplitude and phase balance of the Marchand balun can be tuned, thus effectively improving the efficiency of conversion between single-ended and differential networks in the power amplifier.

**Figure 3:**
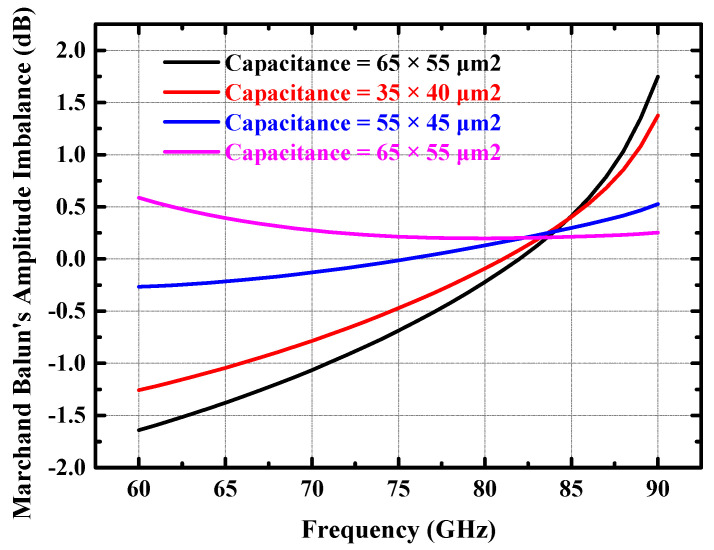
Simulated amplitude balance performance of the input Marchand balun (including RF PAD) at different capacitance values.

**Figure 4:**
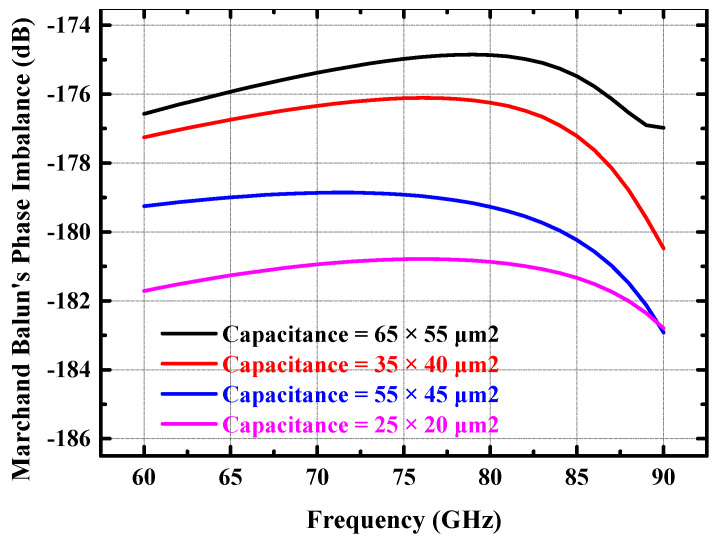
Simulated phase balance performance of the input Marchand balun (including RF PAD) at different capacitance values.

When the tuning capacitor is ideal, the larger the value of the capacitance at the center tap of the secondary coil that forms the balun, the better the common-mode grounding, and the better the balun’s amplitude and phase balance. However, the metal–insulator–metal (MIM) capacitors provided in the silicon-based process exhibit resonance effects in the high-frequency band due to connection parasitics. Figure 3 and Figure 4 show the amplitude and phase balance of the Marchand balun (including RF PAD) obtained from the simulation for different tuning capacitance values. It can be deduced from Figure 3 and Figure 4 that both the amplitude and phase balance of the Marchand balun deteriorate when the tuning capacitor’s value is too large or too small. When the amplitude and phase balance of the differential signal differ significantly, it will seriously degrade the efficiency of the power combining network, which will further degrade the output power and efficiency of the power amplifier. Combining Figure 3 and Figure 4, the size of the Marchand balun’s center capacitor for the input stage was chosen to be 55 × 45 square microns. The corresponding amplitude imbalance of the balun is less than 0.3 dB, and the phase imbalance is less than 1.5 degrees in the 70–85 GHz band.

### 2.2. DC Bias Network with Temperature Compensation

**Figure 5:**
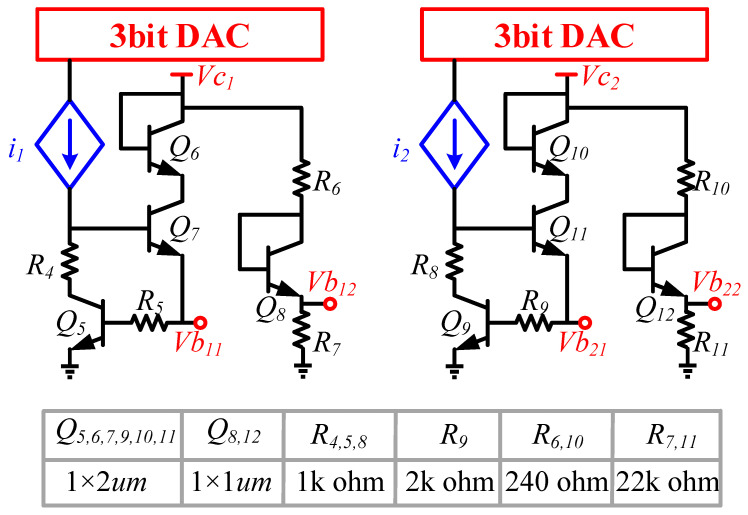
Schematic of the bias network for DA and PA.

**Figure 6:**
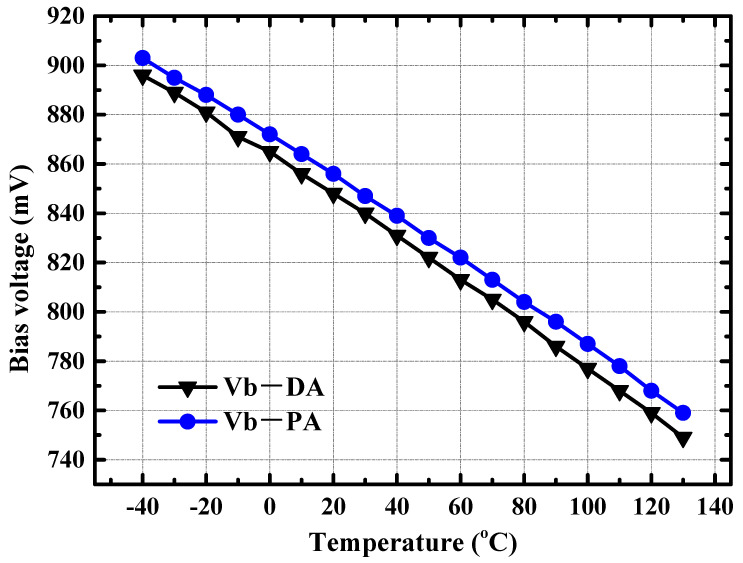
Simulated output bias voltages of the DA/PA at different ambient temperatures.

Power amplifiers for automotive radar applications need to be able to operate properly in ambient temperatures ranging from −40 °C to 125 °C [15]. Traditional power amplifiers use voltage biasing. When the ambient temperature rises or falls, the bias voltage remains fixed, causing the current to abruptly increase in the power amplifier at high temperatures, leading to a sudden change in the transistor’s working state and a significant deterioration in the output power of the power amplifier. To ensure stable RF performance of the power amplifier with changes in ambient temperature [16,17,18,19], we designed an adaptive bias network with temperature compensation characteristics, as shown in Figure 5. Based on the current source controlled by the on-chip integrated 3 bit DAC, the output voltage of this adaptive bias network varies almost linearly with the ambient temperature as shown in Figure 6. The introduction of the DAC is also used for the control of the output power of the power amplifier. By controlling the bits of the DAC, control of the output power of more than 10 dB can be realized. Taking PA as an example, when the ambient temperature is −40 °C, the output voltage of the adaptive bias network is 904 mV. When the ambient temperature rises to 125 °C, the corresponding output bias voltage will be 760 mV. In this way, the PA can maintain a relatively stable current as the ambient temperature changes.

**Figure 7:**
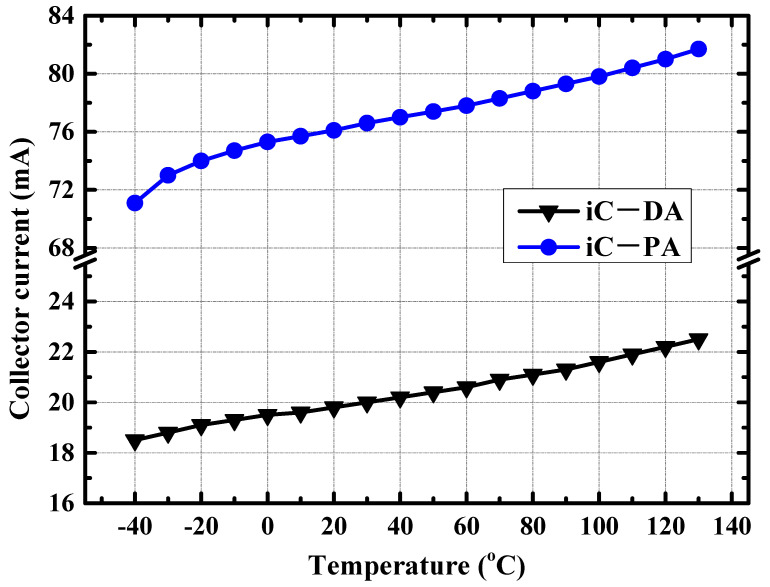
Simulated collector currents of the DA/PA at different ambient temperatures.

**Figure 8:**
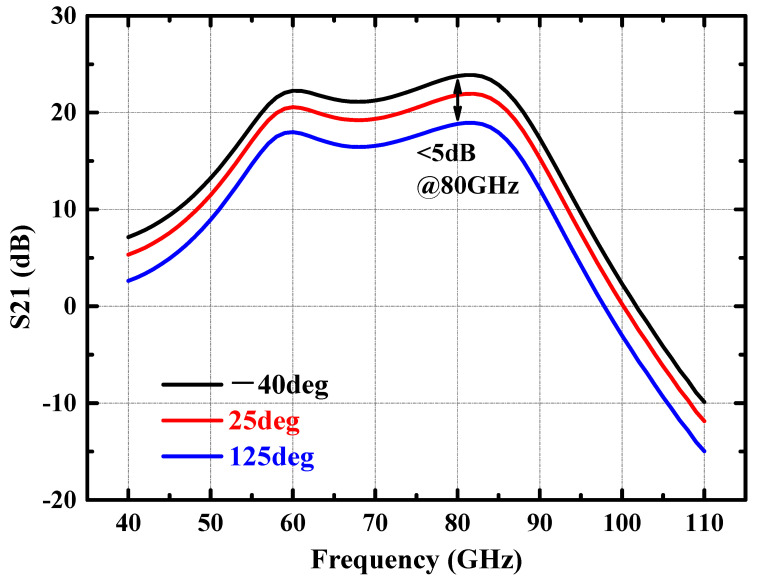
Simulated S21 of the power amplifier at −40 °C, 25 °C, and 125 °C.

**Figure 9:**
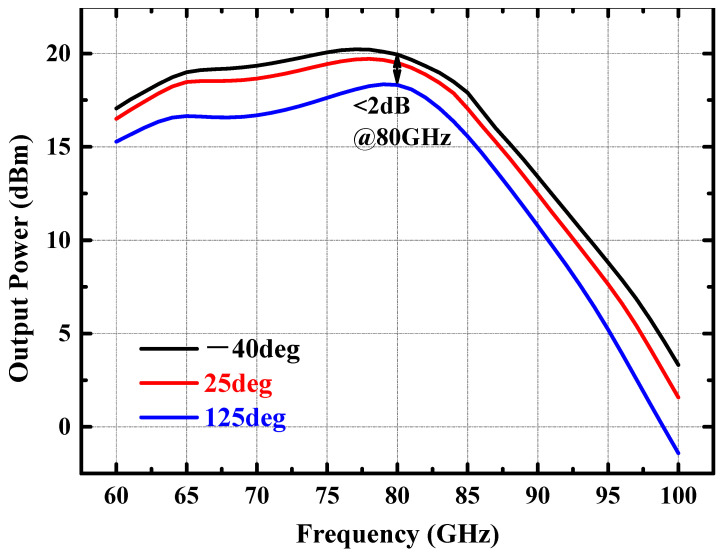
Simulate output power of the power amplifier with 2 dBm input power at −40 °C, 25 °C, and 125 °C.

Benefiting from the adaptive variation of the base bias voltage with the ambient temperature, the collector current of the DA/PA present a relatively stable value with the variation of ambient temperature. As illustrated in Figure 7, when the ambient temperature increases from −40 °C to 125 °C, the collector current of the PA stage only increases from 69.5 mA to 81.8 mA, with a current change percentage of less than 10%. Due to the stable collector current of the power amplifier with temperature changes, the small signal gain and large signal output power of the amplifier remain relatively stable. Figure 8 shows the simulated S21 of the power amplifier at −40 °C, 25 °C, and 125 °C. The small signal gain of the power amplifier fluctuates by less than 3 dB with changes in environmental temperature. When the input power is fixed at 2 dBm, Figure 9 shows the simulated output power of the amplifier in the 70–85 GHz frequency band under different environmental temperature conditions. The output power of the amplifier at room temperature exceeds 17 dBm, and the fluctuation of the output power is less than 2 dBm with temperature changes.

### 2.3. Design of the PA Stage

The use of on-chip multi-path power synthesis scheme can effectively improve the output power of silicon-based 77 GHz power amplifiers, but the parallel of multiple amplifier units leads to a larger chip area [4,5]. Millimeter-wave radar typically requires on-chip integration of multiple transmit channels to support application scenarios such as cascading and angle-scanning, and it is difficult to efficiently integrate multiple channels within the limitations of a half-wavelength when the size of the power amplifier is large.

**Figure 10:**
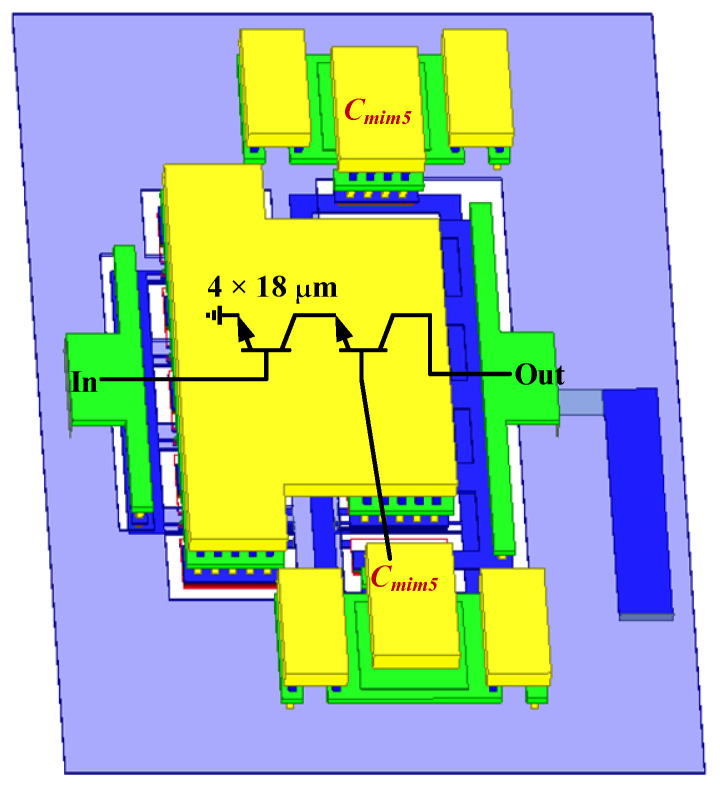
Three-dimensional layout of the PA stage cascode topology transistors.

When designing the power amplifier, we use an on-chip four-way transistor parallel connection scheme in order to ensure that the required chip area is small, and at the same time to increase the output power of the power amplifier as much as possible. The peripheral connection structure of the transistor is shown in Figure 10. In order to ensure the high-efficiency synthesis of the currents between the four parallel transistors, we use a fully symmetrical peripheral connection structure when designing the peripheral connection structure of the transistor. In addition, due to the large size of the transistor, the gain of the transistor is limited, and we introduced two fully symmetric RF grounded capacitors *C*_*mim*5_ at the base of the common-base transistor, as shown in Figure 10, to enhance the gain of the transistor. Based on this design principle, the output power of a single differential power amplifier can exceed 19 dBm. Without using multi-way power synthesis, the output power of the power amplifier can be greatly improved while effectively avoiding a significant increase in the size of the PA.

**Figure 11:**
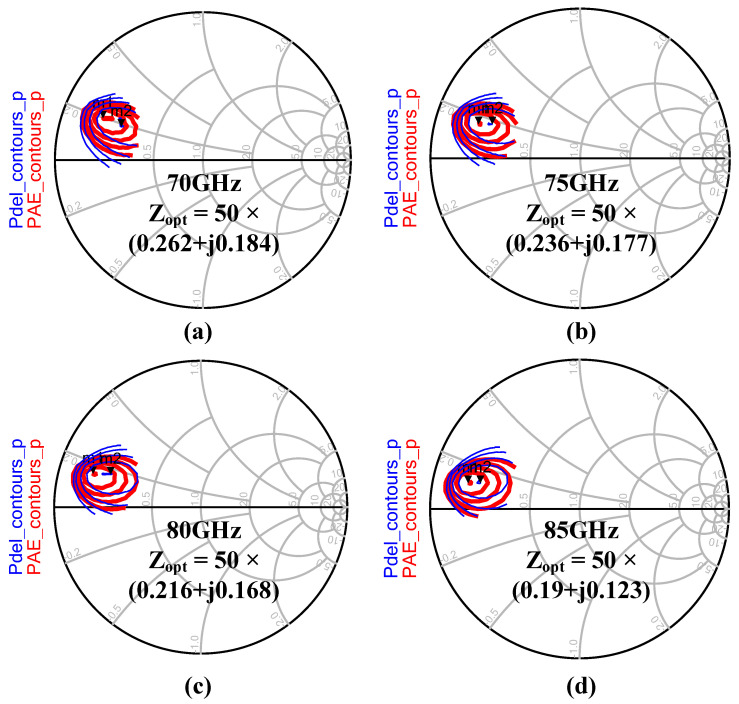
Load-pull simulation of the PA stage with 10 dBm input power at (**a**) 70 GHz, (**b**) 75 GHz, (**c**) 80 GHz, and (**d**) 85 GHz.

Once the transistor sizes of the PA stage were determined, we did a load-pull simulation analysis on the output stage of the power amplifier. Figure 11 shows the simulated load-pull results of the power amplifier at 70 GHz, 75 GHz, 80 GHz, and 85 GHz. The impedance fluctuation corresponding to the optimum output power obtained for this power amplifier is small in frequency. Based on the simulation results of load-pull, the output power matching was accomplished using the Marchand balun designed in Section 2.1 that contains the amplitude and phase-balanced tuning functions.

## 3. Measurement Results

**Figure 12:**
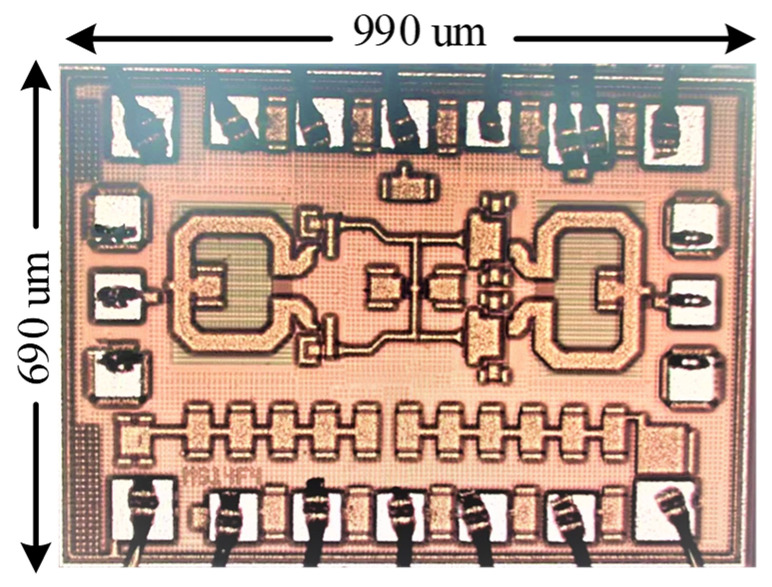
Chip micrograph of the 77 GHz power amplifier.

The power amplifier was realized using a commercial 130 nm SiGe BiCMOS process, and the chip micrograph is shown in Figure 12. The power amplifier has a chip size of only 690 × 990 square microns including DC and RF PADs. The amplifier is biased using a current source controlled by the designed 3-bit DAC with a DC bias voltage of 3.3 V and a static power consumption of about 1 mA per micron emitter size.

**Figure 13:**
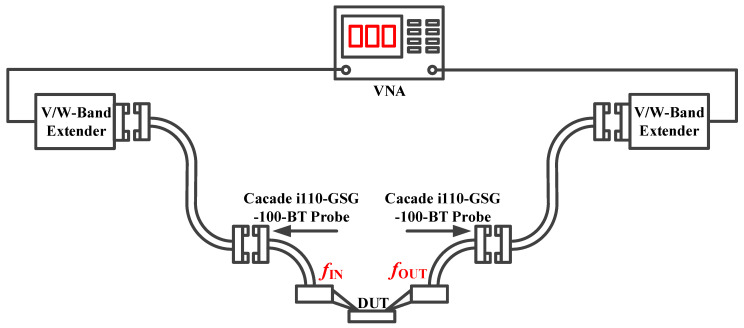
Small signal measurement setup of the power amplifier.

**Figure 14:**
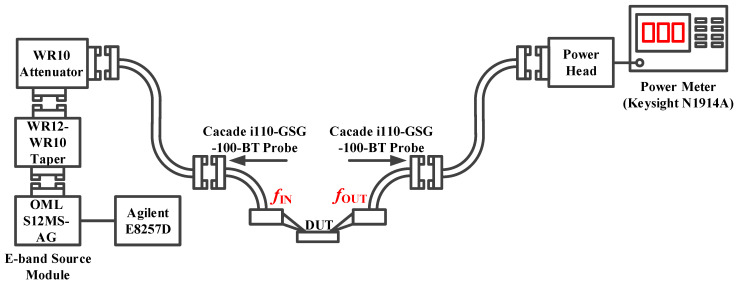
Large signal measurement setup of the power amplifier.

The power amplifier was tested using an on-chip scheme, including small-signal testing and large-signal measurement. The small-signal test platform, shown in Figure 13, contains a Keysight vector network analyzer (VNA), a V(50–75 GHz)/W(75–110 GHz)-band vector network extension, a pair of RF waveguide probes, and a pair of Z-type waveguides. The large-signal test measurement setup is shown in Figure 14, where we focused on the large-signal response in the 70–90 G band. The input RF signal was generated by an Agilent E8257D vector signal source and an OML S12MS-AG frequency multiplier, and the output signal power was measured by a Keysight N1914A power meter.

**Figure 15:**
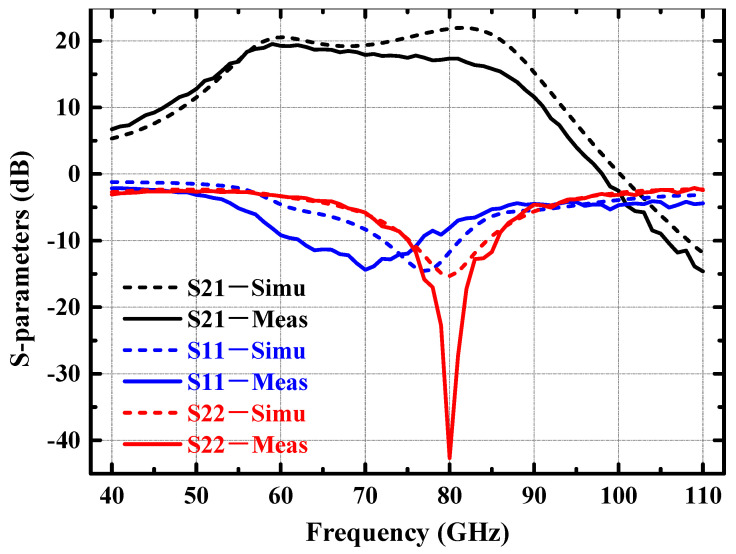
Measured and simulated S-parameters of the 77 GHz power amplifier.

The measured and simulated small-signal performance of the power amplifier are shown in Figure 15. In the 70–85 GHz band, the small-signal gains of the power amplifier obtained from the tests are all more than 16 dB, and the measured S11 and S22 are basically below −6 dB. Comparison of the simulation and measured results of S11 and S22 shows that the EM simulation model is accurate. However, S21 has some deviation at high frequency compared to the simulation results, which may be caused by the inaccuracy of the model of the large-size transistor in the process at high frequency.

**Figure 16:**
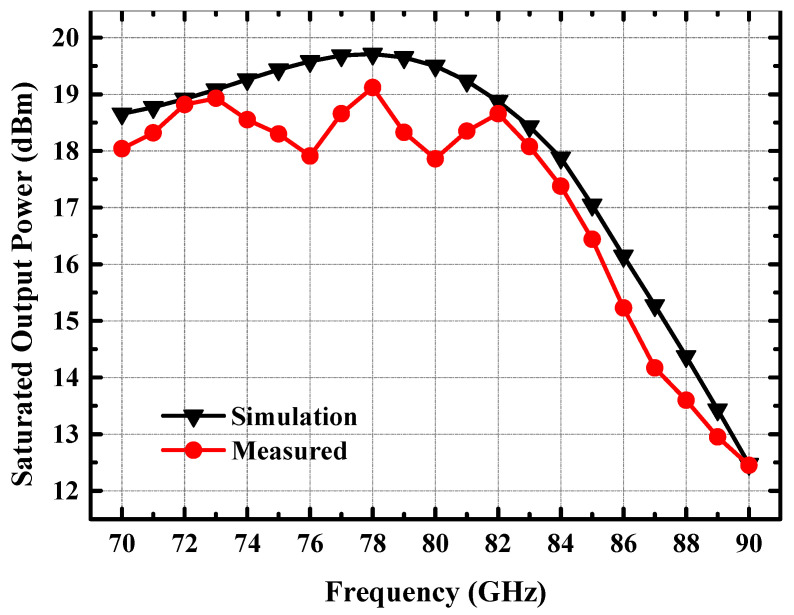
Measured and simulated saturated output power of the 77 GHz power amplifier.

The simulation and measured saturated output power of the power amplifier are given in Figure 16. The measured saturated output power of the amplifier is over 18 dBm in the 70–83 GHz band, and the peak saturated output power of the amplifier reaches 19.1 dBm. The saturated output power obtained from the measurement is lower compared to the simulation, which may be caused by the insufficient gain of the amplifier, resulting in the output power of the DA stage being insufficient to effectively drive the PA stage.

**Figure 17:**
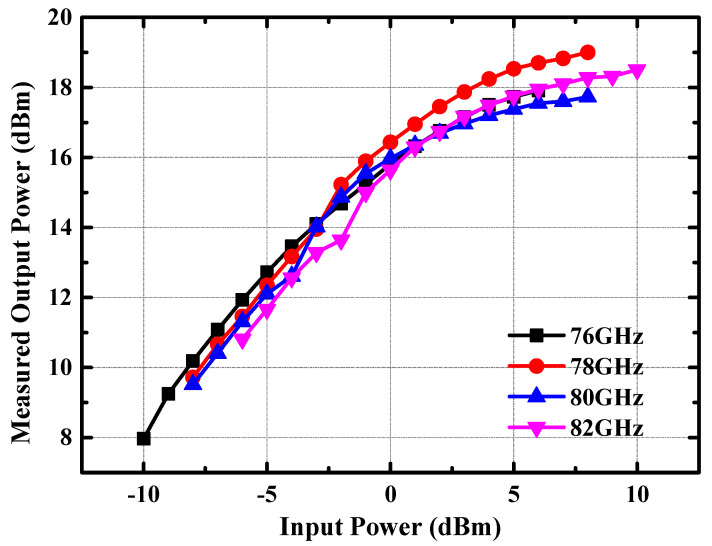
Measured output power versus input power of the 77 GHz power amplifier at 76 GHz, 78 GHz, 80 GHz, and 82 GHz.

In addition, we also conducted tests on the variation of output power of the power amplifier with respect to input power. Figure 17 shows the measured output power versus input power at 76 GHz, 78 GHz, 80 GHz, and 82 GHz. From Figure 17, it can be deduced that with the increase in input power, the output power of the amplifier in the frequency band of 76–81 GHz increases almost linearly. The amplifier’s output power 1 dB compression point was measured to be higher than 16 dBm. Table 1 lists the performance comparison of the power amplifier with previously reported power amplifiers near 77 GHz [20,21,22]. It can be seen that the power amplifier has the minimum chip area when the output power reaches around 20 dBm, which is very competitive in terms of equivalent output power per unit area. Moreover, it can be observed from the comparison table that due to the fact that the power amplifier in this design only integrates a single differential way, the output power of this power amplifier is moderate. However, when normalizing the output power of the reported power amplifiers to each way, this power amplifier has the highest output power, as shown in the penultimate row of Table 1.

## 4. Conclusions

In this paper, a differential power amplifier chip is designed and realized based on SiGe process with an operating frequency covering a 76–81 GHz band. The inputs and outputs of the power amplifier are single-ended for ease of testing and application. At the input and output of the power amplifier, we introduce a Marchand balun to realize the conversion between single-ended and differential signals. In order to effectively enhance the amplitude and phase balance of the Marchand balun, a capacitor that achieves amplitude and phase balance between the balun’s differential ports is introduced at the center tap that constitutes the balun’s secondary coil, which enhances the performance of the balun while effectively increasing the output power of the power amplifier. In addition, we designed an on-chip integrated temperature compensation network to ensure that the power amplifier exhibits stable output power and gain characteristics over ambient temperature variations (from −40 °C to 125 °C). In the design of the temperature compensated network, a 3-bit DAC-controlled current source is introduced, allowing the amplifier to have an output power tuning range of more than 10 dB. The measured results show that the power amplifier has a peak output power of more than 19 dBm, which is promising for applications in millimeter-wave radar and other fields.

## Figures and Tables

**Table 1:** Comparison with previous silicon-based PAs at around 77 GHz.

	[4]	[5]	[6]	[20]	[21]	[22]	This Work
Process	40 nm CMOS	22 nm FDSOI	120 nm SiGe	40 nm CMOS	130 nm SiGe	120 nm SiGe	120 nm SiGe
Frequency [GHz]	77	77	77	73	71–76	77	77
Supply voltage [V]	1.1	2	2	1.8	3.3	2	3.3
Combination way	4	1	4	4	8	2	1
Topology	Common source	Cascode	Common emitter	Common source	-	Common emitter	Cascode
Gain [dB]	19.4	17.8	22	25.3	46 (TX)		17.5
Psat [dBm]	19.7	17.8	24	22.6	25	19.5	19.1
Psat/way [dBm]	12.7	17.8	18	16.6	16	16.5	19.1
Area [mm^2^]	0.75	-	3.75	>0.75	5.4	1.46	0.68

## Data Availability

Data are contained within the article.
